# Combined surface functionalization of MSC membrane and PDA inhibits neurotoxicity induced by Fe_3_O_4_ in mice based on apoptosis and autophagy through the ASK1/JNK signaling pathway

**DOI:** 10.18632/aging.204884

**Published:** 2023-07-19

**Authors:** Yang Li, Te Liu, Xiuying Li, Modi Yang, Tianxin Liu, Jindian Bao, Miao Jiang, Lingling Hu, Yuzhuo Wang, Pu Shao, Jinlan Jiang

**Affiliations:** 1 Scientific Research Center, China-Japan Union Hospital of Jilin University, Changchun, Jilin, China; 2 Yibin Jilin University Research Institute, Jilin University, Yibin, Sichuan, China; 3 Department of Orthopeadics, China-Japan Union Hospital of Jilin University, Changchun, Jilin, China; 4 Jilin University School of Public Health, Changchun, Jilin, China; 5 Department of Orthodontics, School and Hospital of Stomatology, Jilin University, Changchun, Jilin, China

**Keywords:** Fe3O4, nanoparticles, stem cell membrane, apoptosis, autophagy

## Abstract

The extensive utilization of iron oxide nanoparticles in medical and life science domains has led to a substantial rise in both occupational and public exposure to these particles. The potential toxicity of nanoparticles to living organisms, their impact on the environment, and the associated risks to human health have garnered significant attention and come to be a prominent area in contemporary research. The comprehension of the potential toxicity of nanoparticles has emerged as a crucial concern to safeguard human health and facilitate the secure advancement of nanotechnology. As nanocarriers and targeting agents, the biocompatibility of them determines the use scope and application prospects, meanwhile surface modification becomes an important measure to improve the biocompatibility. Three different types of iron oxide nanoparticles (Fe_3_O_4_, Fe_3_O_4_@PDA and MSCM-Fe_3_O_4_@PDA) were injected into mice through the tail veins. The acute neurotoxicity of them in mice was evaluated by measuring the levels of autophagy and apoptosis in the brain tissues. Our data revealed that iron oxide nanoparticles could cause nervous system damage by regulating the ASK1/JNK signaling pathway. Apoptosis and autophagy may play potential roles in this process. Exposure to combined surface functionalization of mesenchymal stem cell membrane and polydopamine showed the neuroprotective effect and may alleviate brain nervous system disorders.

## INTRODUCTION

In recent years, iron oxide nanoparticles, which are with magnetic responsiveness, have made extensive progress in the application of nanomedicine [1]. Magnetic nanoparticles have unique properties, such as small size effect, surface effect, quantum size effect and unique magnetism, which show the significant application value in many fields [1, 2].

Iron oxide nanoparticles can be used in clinical practice [3]. They are also widely applied in medical care and life sciences, such as magnetic-assisted drug delivery, magnetic resonance imaging contrast agents, photothermal therapy and tissue engineering, considering their strong drug-loading capacity, good biocompatibility and high targeting delivery efficiency. As new nanocarriers and targeting agents, the biocompatibility determines their application scope and prospect. Therefore, surface modification becomes an important method to change their biocompatibility.

However, exposure to iron oxide nanoparticles can lead to significant toxicity, such as formation of apoptotic bodies, inflammation, impaired mitochondrial function, high levels of reactive oxygen species (ROS), increased micronuclei (indicators of total chromosome damage, indicators of genotoxicity) and chromosome concentration. Iron oxide nanoparticles showed cytotoxicity and could induce apoptosis in hepatocellular carcinoma cells and non-small-cell lung cancer cells [4]. Iron oxide nanoparticles could weaken the motor coordination and spatial memory in animals. Exposure to iron oxide nanoparticles greater than 20 μg/ml induced a significant decrease in the viability of human fetal nerve precursor cell [5]. Animal experiments have confirmed that the accumulation of titanium dioxide nanoparticles in the hippocampus could lead to apoptosis of hippocampus cell and deterioration of spatial recognition memory, resulting in functional neurotoxicity [6]. Ag, Cu or Al/Al_2_O_3_ nanoparticles could also destroy blood-brain barrier, meanwhile induce brain edema [7–10].

These findings suggested that exposure to Fe_3_O_4_ nanoparticles (Fe_3_O_4_ NPs) and other nanoparticles might lead to brain injury, but the specific pathogenesis remains unclear. Therefore, it is urgent for scholars to clarify its toxic mechanism in the nervous system and determine how to reduce the toxicity of nanoparticles and improve their biocompatibility. Stem cells can treat various diseases because of their ability to repair tissue and cell damage, replace damaged cells and stimulate the regeneration of their own cells [11, 12].

Given the good biocompatibility and biodegradability, polydopamine (PDA) has been applied to nanoparticles camouflage in diverse biomedical area [13]. Studies have demonstrated that Fe_3_O_4_ modified with PDA still exhibits some biotoxicity, even though its biocompatibility is significantly improved [14–16]. Therefore, a new method for Fe_3_O_4_ modification is urgently needed for clinical application. Mesenchymal stem cells (MSCs) are highly targeted and biocompatible [17]. By separating cell membranes with unique biological properties and constructing a nanoparticle system camouflaged with the natural cell membranes, the ability of nanoparticles to avoid detection and eliminate disease and to improve the long circulation of blood can be improved, and the biological distribution of nanoparticles *in vivo* can be improved.

This study uses cellular autophagy and apoptosis as an entry point to further clarify the toxicity and biocompatibility of Fe_3_O_4_ NPs camouflaged by MSC membrane and PDA in the nervous system, and explore whether they are involved in regulating apoptosis and autophagy through the apoptosis signal-regulating kinase 1 (ASK1)/c-Jun NH2-terminal kinase (JNK) signaling pathway, providing a scientific basis for addressing the application and safety in clinical therapy.

## MATERIALS AND METHODS

### Preparation of NPs (Fe_3_O_4_, Fe_3_O_4_@PDA and MSCM-Fe_3_O_4_@PDA) with three different characteristics

Fe_3_O_4_ NPs and Fe_3_O_4_@PDA NPs were synthesized through the thermal decomposition method, as described formerly [15]. The human umbilical cord was taken from a full-term cesarean delivery at the China-Japan Union Hospital of Jilin University. Patients were informed and consented to the donation in advance. Human umbilical mesenchymal stem cells (HUMSCs) were obtained by primary culture of the tissue. HUMSCs are harvested and washed with PBS thrice. Cells are suspended in a hypotonic lysing buffer consisting of 1 mM NaHCO_3_, 0.2 mM EDTA and 1 mM PMSF at 4°C. The suspended cell solution was ground using a Dounce homogenizer approximately 10 times. The suspended solution is centrifuged at 3000 g for 5 min at 4°C, and the resulting supernatant is centrifuged at 15000 g for 30 min at 4°C. HUMSCs, which were acquired from American Type Culture Collection (Manassas, VA, USA), and HUMSCs membranes were mixed with Fe_3_O_4_@PDA at a polymer-to-membrane protein weight ratio of 2:1 and coextruded 10 times through a 200-nm pore size polycarbonate membrane. NPs of Fe_3_O_4_@PDA camouflaged with HUMSC membranes were obtained and abbreviated as MSCM-Fe_3_O_4_@PDA.

NPs were prepared into a granular suspension with 10 mg/mL Fe^3+^ concentration in normal saline. After shaking and mixing, the suspension was treated by ultrasound for 1 min (6 secs each time, 10 seconds interval). The suspension was sterilized by 0.45-μm membrane filtration and preserved at 4°C.

### Characterization of NPs

Transmission electron microscopy (TEM) was used to observe the morphology of Fe_3_O_4_, Fe_3_O_4_@PDA and MSCM-Fe_3_O_4_@PDA. Fe_3_O_4_@PDA nanoparticles camouflaged by HUMSC membranes can be better observed by 1% uranyl acetate staining. The size and zeta potential of three nanoparticles were determined by dynamic light scattering (DLS) (Wyatt Technology Corporation, Goleta, CA, USA). To characterize the protein components of nanoparticles, all samples were analyzed by sodium dodecyl sulfate polyacrylamide gel electrophoresis (SDS-PAGE). After electrophoresis, the protein strips were colored with Coomassie brilliant blue solution and decolorized with glacial acetic acid. The specific surface markers of HUMSC on Fe_3_O_4_@PDA, HUMSC lysate, HUMSC membrane vesicles and HUMSC membranes camouflaged with Fe_3_O_4_@PDA were examined by western blot, including Na^+^-K^-^ ATPase (Abcam, 1:1000), GAPDH (Abcam, 1:5000), CXCR4 (Abcam, 1:1000) and other signature proteins.

### Animals and exposure

ICR mice weighing (20 ± 2) g were selected. Mice were randomly divided into saline control group, MSCM-Fe_3_O_4_@PDA, Fe_3_O_4_@PDA and Fe_3_O_4_ exposure groups with 12 mice in each group. The dosage of Fe^3+^ was 45 mg/kg.kw/d (1/10 LD_50_) for 4 weeks.

### Collection of tissues

The whole mice were weighed and anesthetized with isoflurane after a 24-hour fasting. The blood was drawn from each mouse’s heart by Vacutainer and centrifuged for 10 minutes at a speed of 5000 rpm after a flagellated layer of 4°C. Brain tissues were removed and then stored in liquid nitrogen.

### Detection of biological index

Interleukin-1β (IL-1β), interleukin-6 (IL-6), interleukin-10 (IL-10), interleukin-13 (IL-13), interferon γ (IFN-γ), tumor necrosis factor-α (TNF-α) and macrophage inflammatory protein-2 (MIP-2) levels in serum and brain tissues were assayed using an ELISA kit (R&D Systems, Minneapolis, MN, USA) in strict accordance with the manufacturer’s instructions.

Levels of ROS, superoxide dismutase (SOD), malondialdehyde (MDA) and glutathione Peroxidase (GSH-Px) were detected using respective reagent kits. These commercial kits were purchased from Nanjing Jiancheng Bioengineering Institute (Nanjing, China). All assays were performed according to the instructions.

### TUNEL assay of apoptosis in brain tissue

The tissues were soaked in 10% (v/v) formalin, embedded in paraffin, and then cut into 5-μm sections with a microtome (Leica RM2145, Germany). The sections were dewaxed by sequential immersion in xylene and gradually increasing concentrations of alcohol. TdT-mediated dUTP Nick-End Labeling (TUNEL) was used for detection. The experimental process was operated according to the requirements of the kit (Roche, USA). The image-pro plus system analyzes the total positive cells number observed under optical microscopy (10 randomly selected fields, 400× magnification).

### Assessment of brain neuron autophagy by TEM

After removing the brain tissue of mice, the parietal lobe was removed and fixed in 2.5% glutaraldehyde. The samples were washed, embedded, sliced and stained. Parietal lobe neuron autophagy was observed by TEM.

### RNA extraction and qRT-PCR analysis

Trizol (TaKaRa, Japan) reagent was used to extract Total RNA. Briefly, 500 ng of RNA was reverse transcribed into cDNA, and SYBR Premix Ex TaqII (TaKaRa, Japan) was used based on the manufacturer’s instructions. PCRs were performed as follows: 45 cycles at 95°C for 20 s and 60°C for 20 s. *Bcl-2, Bax, Caspase-3*, *Caspase-8, Caspase-9, LC3, p62, Beclin1, ASK1, JNK* and *β-actin* were selected to be the candidate genes, and *β-actin* was the internal reference. The primer sequences used to amplify each gene are shown in Table 1.

**Table 1:** Gene-specific forward and reverse primer sequence.

**Target gene**	**Direction**	**Sequence**
*Caspase-3*	Forward	AGGAGCAGCTTTGTGTGTGTG
	Reverse	CCAGTCAGACTCCGGCAGTAG
*Caspase-8*	Forward	CACAAGAAGCAGGAGACCATCGAG
	Reverse	GCAGTCTAGGAAGTTGACCAGCAG
*Caspase-9*	Forward	CTGGGTCTCGGCGGGATCAG
	Reverse	AGCAGGAGATGAAGAGAGGAAGGG
*Bcl-2*	Forward	CAGAGGGGCTACGAGTGGGATG
	Reverse	TGGGTTGCTCTCAGGCTGGAAG
*Bax*	Forward	TGCTGACGTGGACACGGACTC
	Reverse	AGCAAAGTAGAAGAGGGCAACCAC
*LC3*	Forward	GCCTTCTTCCTGCTGGTCAACC
	Reverse	GCGTAGACCATGTAGAGGAATCCG
*p62*	Forward	GATCCGCCGCTTCAGCTTCT
	Reverse	CTCAGCAGCCTCTCGCAGG
*Beclin1*	Forward	GTCTAAGGCGTCCAGCAGCAC
	Reverse	GTCCAGGATCTTGAAGCTCGTGTC
*ASK1*	Forward	AGTGGCACTTGGATTCAGTGATGG
	Reverse	GAGCAACAGGAAGCGGTCGAG
*JNK*	Forward	CCACCGCCATTCAGACTGTG
	Reverse	TTGTCACTGGGTGAGCCTGA
*β-actin*	Forward	CACCCGCGAGTACAACCTTC
	Reverse	CCCATACCCACCATCACACC

### Immunohistochemistry

Brain tissues were immobilized in 4% paraformaldehyde solution for 24 hours and then dehydrated in 25% sucrose solution. The above specimens were embedded, sliced at 6 μm and stored at −20°C. The frozen sections were washed with PBS after rewarming. Serum was blocked and incubated with antibodies, including anti-rabbit Bcl-2 (Abcam, 1:250), anti-rabbit Bax (Abcam, 1:250), anti-rabbit cleaved Caspase-9 (Cell Signaling, 1:800), anti-rabbit cleaved Caspase-9 (Cell Signaling, 1:500), anti-rabbit cleaved Caspase-3 (Abcam, 1:100), anti-rabbit LC3 (Abcam, 1:2000), anti-rabbit p62 (Proteintech, 1:50), anti-rabbit Beclin1 (Abcam, 1:100), anti-rabbit ASK1 (Abcam, 1:50), rabbit anti-phospho-ASK1 (Thermo Fisher, 1:50), anti-mouse JNK (Proteintech, 1:500), rabbit anti-phospho-JNK (Abcam, 1:100), and anti-mouse β-actin (Proteintech, 1:2000), for 16~18 hours at 4°C. Each section was washed with PBS and then incubated with the proper secondary antibody (Abcam, 1:5000) for 30 minutes. It was visualized by staining with 3,3′-diaminobenzidine (DAB) (Bioss Biotechnology Company, Woburn, MA, USA) kit, and then observed under an optical microscope and photographed. Image visualization used Image-Pro Plus 6.0 based on the integral optical density, which reflects the change of optical density and area of the positive substance, i.e., the total antigen level.

### Western blot

RIPA lysis buffer was used to extract proteins from brain tissues, and then the bicinchoninic acid protein assay kit (Beyotime, China) was used to determine the protein concentration. Equal amounts of protein were subjected to 10% SDS-PAGE prior to transfer onto PVDF membranes (Millipore). After being blocked with 5% nonfat milk, the membranes were then incubated with proper antibodies, including anti-rabbit Bcl-2 (Abcam, 1:1000), anti-rabbit Bax (Abcam, 1:1000), anti-rabbit cleaved Caspase-9 (Cell Signaling, 1:1000), anti-rabbit cleaved Caspase-8 (Cell Signaling, 1:1000), anti-rabbit cleaved Caspase-3 (Abcam, 1:500), anti-rabbit LC3 (Abcam, 1:3000), anti-rabbit p62 (Proteintech, 1:1000), anti-rabbit Beclin1 (Abcam, 1:1000), anti-rabbit ASK1 (Abcam, 1:500), rabbit anti-phospho-ASK1 (Thermo Fisher, 1:500), anti-mouse JNK (Proteintech, 1:3000), rabbit anti-phospho-JNK (Abcam, 1:1000), and anti-mouse β-actin (Proteintech, 1:2000). The mouse β-actin was used as internal reference protein. After being washed, membranes were then incubated with anti-rabbit IgG (H+L) (CST, 1:15000) or anti-mouse IgG (CST, 1:15000) fluorescent secondary antibodies. Densiometric scan and Image J were used to quantify the relative protein levels.

### Statistics analysis

SPSS 22.0 statistical software (SPSS Inc., Chicago, IL, USA) was selected for the statistical analysis. Based on the variance normality, the data were listed as the mean ± standard deviation. The differences among groups were tested through one-way ANOVA followed by Tukey–Kramer post hoc test for multiple comparisons. *p* < 0.05 was considered to be statistically significant.

### Availability of data and materials

The datasets used to support the findings of this study are included in the article.

## RESULTS

### Characterization of NPs

**Figure 1:**
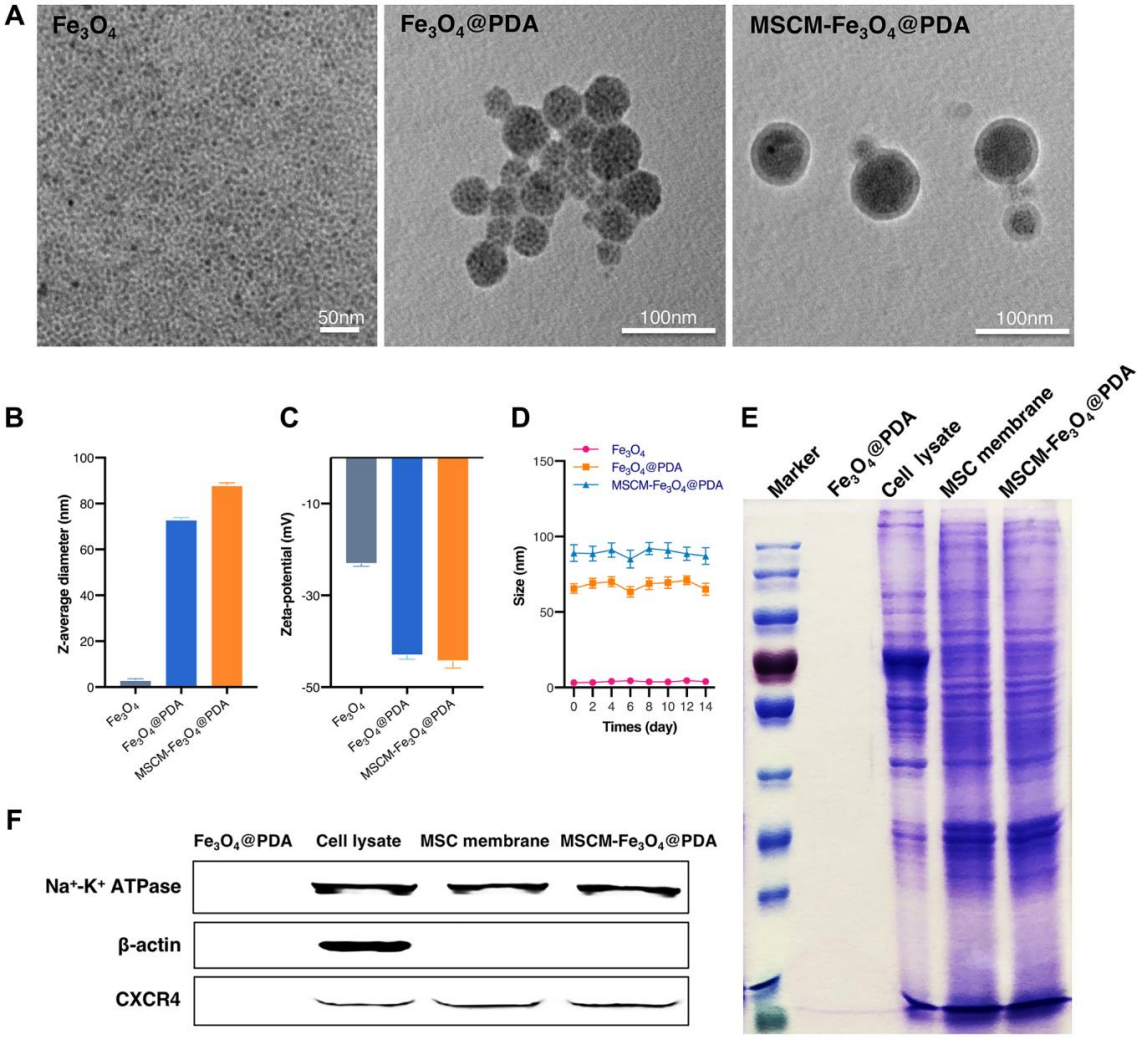
**Characterization of Fe_3_O_4_, Fe_3_O_4_@PDA and MSCM-Fe_3_O_4_@PDA NPs.** (**A**) TEM images of NPs. (**B**) DLS of NPs. (**C**) Zeta potential of NPs. (**D**) Stability analysis of NPs. (**E**) SDS-PAGE protein analysis of Fe_3_O_4_@PDA, Cell lysate, MSC membrane and MSCM-Fe_3_O_4_@PDA. (**F**) Membrane protein expressions of Fe_3_O_4_@PDA, Cell lysate, MSC membrane and MSCM-Fe_3_O_4_@PDA.

TEM showed that Fe_3_O_4_, Fe_3_O_4_@PDA and MSCM-Fe_3_O_4_@PDA all displayed a homogeneous spherical structure. After coated with HUMSC membranes, NPs revealed a clear spherical core-shell structure, indicating that the HUMSC membranes were successfully coated on Fe_3_O_4_@PDA (Figure 1A). According to the DLS in Figure 1B, our consequences showed that the average hydrodynamic diameter of Fe_3_O_4_ was 3.2 ± 0.46 nm. In comparison with Fe_3_O_4_@PDA, the average hydrodynamic diameters of MSCM-Fe_3_O_4_@PDA increased from 73.1 ± 0.8 to 88.1 ± 0.9 nm. The increase of 15 nm in diameter might result from the thickness of HUMSC membrane formed by the lipid bilayer. Figure 1C showed the Zeta potential of the three nanomaterials. The surface Zeta potential of the Fe_3_O_4_ NPs was −23.2 ± 0.46 mV. In comparison with Fe_3_O_4_@PDA nanoparticles, the surface Zeta potential of MSCM-Fe_3_O_4_@PDA changed from −43.1 ± 0.8 mV to −44.4 ± 1.4 mV, which might be because the HUMSCs film shields the surface charge of nanoparticles. The three curves shown in Figure 1D did not show large fluctuations. No obvious change in size is noted for these three types of nanoparticles during the observation period. The long-term stability of Fe_3_O_4_, Fe_3_O_4_@PDA and MSCM-Fe_3_O_4_@PDA nanoparticles was confirmed.

MSCM-Fe_3_O_4_@PDA NPs membrane protein characterization results were presented in Figure 1E. After SDS-PAGE electrophoresis, we found that the HUMSC membrane surface of the MSCM-Fe_3_O_4_@PDA NPs showed almost the same protein composition as the HUMSC membrane. This finding indicated that HUMSC membrane protein was completely retained during the preparation of MSCM-Fe_3_O_4_@PDA NPs. In addition, we used western blotting to detect the expression of membrane protein CXC-chemokine receptor 4 (CXCR4) located on the HUMSC membrane surface, and β-actin and Na^+^-K^+^-ATPase served as internal reference proteins. As shown in Figure 1F, Na^+^-K^+^-ATPase and CXCR4 protein bands were observed on the surface of MSCM-Fe_3_O_4_@PDA NPs, HUMSC lysates and HUMSC membranes. Furthermore, β-actin band was clearly observed in HUMSC lysate but almost absent on the surface of MSCM-Fe_3_O_4_@PDA NPs and HUMSC membranes. This finding indicated that the purity of the extracted HUMSCs membrane was very high and further confirmed that the surface of Fe_3_O_4_@PDA NPs was successfully camouflaged by the HUMSCs membrane.

### Effects of NPs on body weight

The body weight change in experimental animals is a sensitive and objective index that reflects the influence of chemicals on the general condition and growth of experimental animals. Studies *in vivo* have demonstrated that animals exposed to nanoparticles may be affected by toxicity, resulting in weight loss or slower weight gain. In our study, mice treated with nanoparticles appeared less active, moved more slowly, and had untidy and lusterless fur. The body weight of mice in each group increased with time, and no significant difference was noted between these groups (Table 2). It is possible that the NPs exposure dose was low, so there was no significant toxic effect on body weight.

**Table 2:** Body weights of mice treated with nanoparticles during the administration period (mean ± SE, g).

**Groups**	***n***	**Time (Weeks)**			
		**1**	**2**	**3**	**4**
Control	12	25.23 ± 1.25	27.65 ± 1.23	29.72 ± 1.09	30.85 ± 1.44
MSCM-Fe_3_O_4_@PDA	12	25.18 ± 1.70	27.27 ± 1.40	29.52 ± 1.05	30.42 ± 1.67
Fe_3_O_4_@PDA	12	24.90 ± 1.46	27.04 ± 0.89	29.37 ± 0.95	29.86 ± 1.71
Fe_3_O_4_	12	24.75 ± 0.88	27.12 ± 1.35	29.26 ± 1.46	29.53 ± 2.11

### Effects of NPs on inflammatory factors in mice

**Figure 2:**
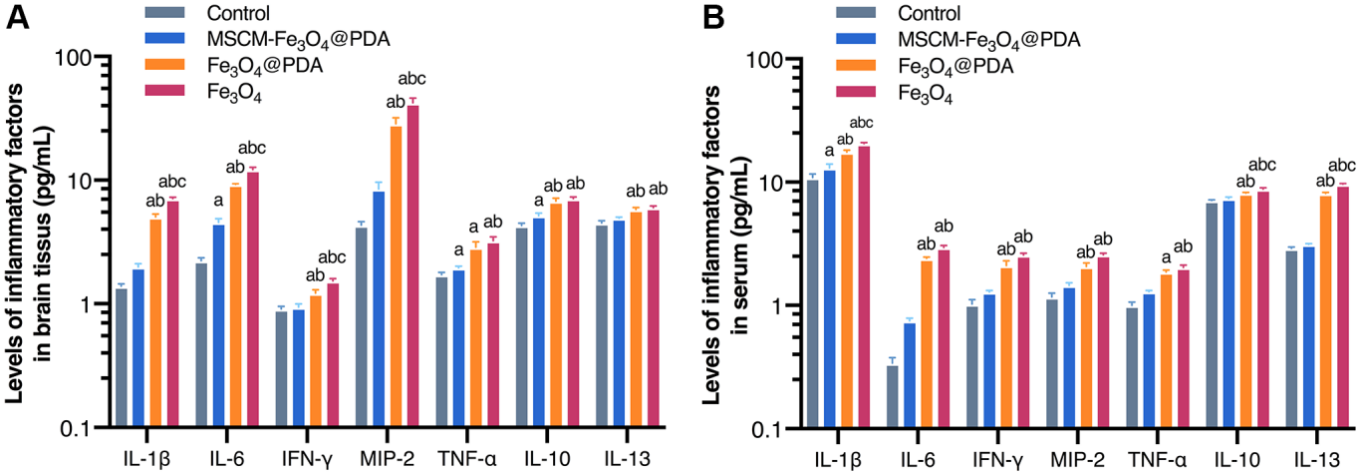
Levels of inflammatory factors in brain tissues (**A**) and serum (**B**) after exposure to MSCM-Fe_3_O_4_@PDA, Fe_3_O_4_@PDA and Fe_3_O_4_ NPs. ^a^*p* < 0.05 when compared with control, ^b^*p* < 0.05 when compared with MSCM-Fe_3_O_4_@PDA, ^c^*p* < 0.05 when compared with Fe_3_O_4_@PDA.

IL-1β, IL-6, IL-10, IL-13, IFN-γ, TNF-α, and MIP-2 levels were detected in brain tissues and serum (Figure 2A and 2B). In our study, we found that the levels of IL-1β, IL-6, IFN-γ, MIP-2, TNF-α, IL-10 and IL-13 in brain tissues and blood were similar. The levels in the Fe_3_O_4_@PDA and Fe_3_O_4_ groups were increased significantly compared with the control and MSCM-Fe_3_O_4_@PDA groups (*p* < 0.05), while IL-6 and TNF-α levels in the MSCM-Fe_3_O_4_@PDA-treated group were increased significantly compared with controls (*p* < 0.05).

### Effects of NPs on oxidative stress in mice brain tissues

**Figure 3:**
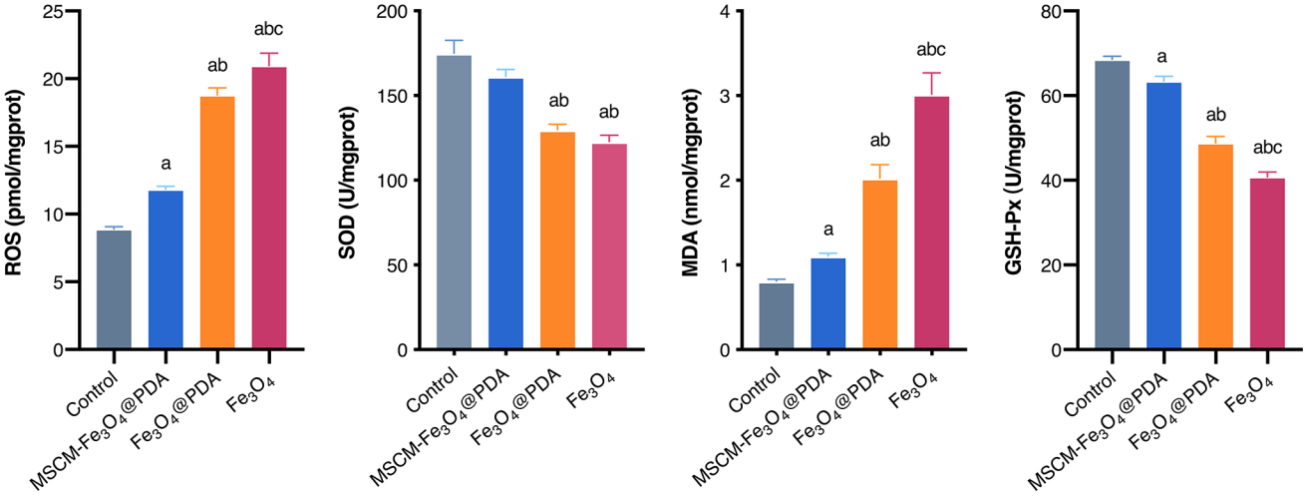
**Levels of ROS, SOD, MDA, and GSH-Px after exposure to MSCM-Fe_3_O_4_@PDA, Fe_3_O_4_@PDA and Fe_3_O_4_ NPs.**^ a^*p* < 0.05 when compared with control, ^b^*p* < 0.05 when compared with MSCM-Fe_3_O_4_@PDA, ^c^*p* < 0.05 when compared with Fe_3_O_4_@PDA.

As shown in Figure 3, ROS and MDA levels in NP groups in brain tissues were significantly increased. In comparison with the control group, the level in the MSCM-Fe_3_O_4_@PDA group was significantly increased. Compared with the MSCM-Fe_3_O_4_@PDA group and the control group, the level in Fe_3_O_4_@PDA group increased. The levels in the Fe_3_O_4_ group were significantly increased compared with the other three groups (*p* < 0.05). SOD levels in Fe_3_O_4_@PDA and Fe_3_O_4_ groups were decreased, while GSH-Px levels in all nanoparticle-treated groups were decreased compared to the control (*p* < 0.05).

### Effects of NPs on apoptosis in brain tissues of mice

**Figure 4:**
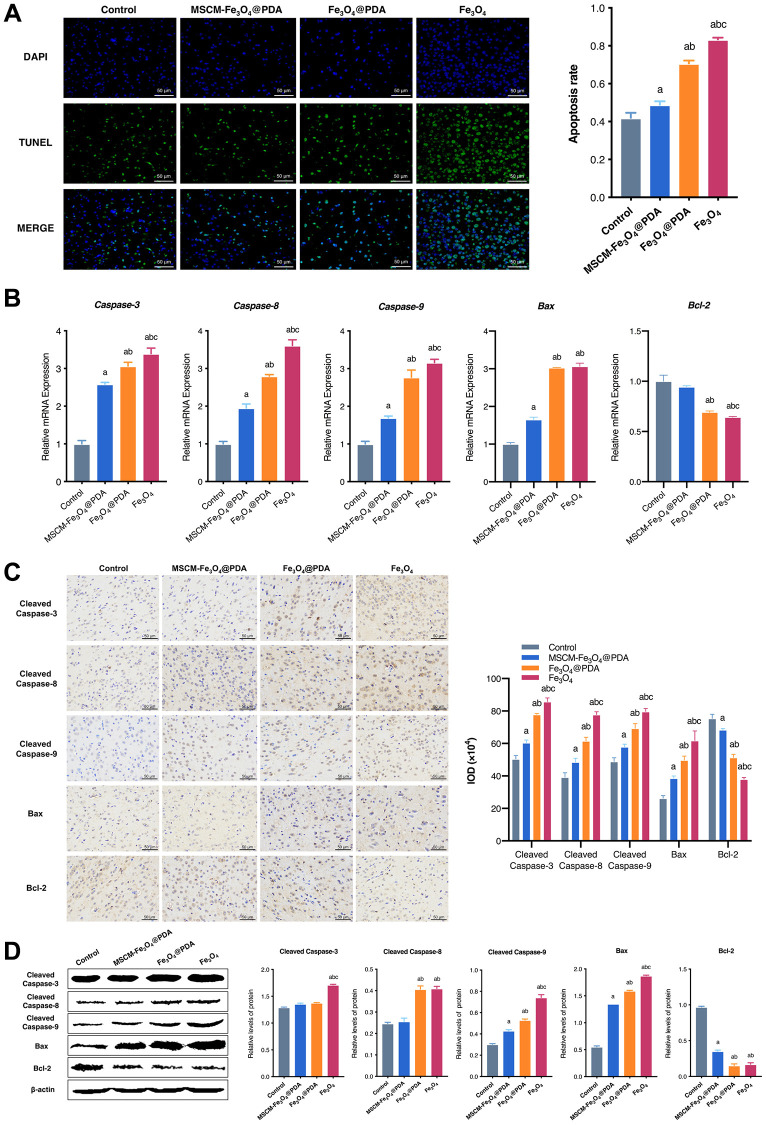
**Effects of MSCM-Fe_3_O_4_@PDA, Fe_3_O_4_@PDA and Fe_3_O_4_ NPs on apoptosis.** (**A**) TUNEL staining to observe the apoptosis of neuron cells in mice brain. (**B**) Detection of Caspase-3, Caspase-8, Caspase-9, Bax and Bcl-2 mRNA levels by RT-qPCR. (**C**) The representative pictures and statistical chart of Cleaved Caspase-3, Cleaved Caspase-8, Cleaved Caspase-9, Bax and Bcl-2 protein by immunohistochemistry. (**D**) Detection of Cleaved Caspase-3, Cleaved Caspase-8, Cleaved Caspase-9, Bax and Bcl-2 protein by western blot. ^a^*p* < 0.05 when compared with control, ^b^*p* < 0.05 when compared with MSCM-Fe_3_O_4_@PDA, ^c^*p* < 0.05 when compared with Fe_3_O_4_@PDA.

To reveal the neurotoxicity of three nanoparticles, we detected apoptosis levels in brain tissues in mice. TUNEL staining results revealed that cell apoptosis was notably induced by Fe_3_O_4_@PDA and Fe_3_O_4_, and these effects were significantly attenuated by MSCM-Fe_3_O_4_@PDA (Figure 4A). To further confirm this result, the expression of apoptosis-related proteins and genes, including Caspase-3, Cleaved Caspase-3, Caspase-8, Cleaved Caspase-8, Caspase-9, Cleaved Caspase-9, Bax and Bcl-2, were examined.

In the current study, qRT-PCR (Figure 4B) showed that MSCM-Fe_3_O_4_@PDA significantly inhibited the increases in Caspase-3, Caspase-8, Caspase-9, and Bax levels induced by Fe_3_O_4_@PDA and Fe_3_O_4_. However, Bcl-2 levels in control and MSCM-Fe_3_O_4_@PDA were significantly increased. In brain tissues of mice, we also used immunohistochemistry (Figure 4C) and western blot (Figure 4D) to detect the expression of apoptosis-related proteins. The results showed that the levels of Cleaved Caspase-3, Cleaved Caspase-8, Cleaved Caspase-9, and Bax in the Fe_3_O_4_ group were significantly increased compared with the other three groups, whereas the Bcl-2 level was reduced. Compared with the Fe_3_O_4_@PDA group, the MSCM-Fe_3_O_4_@PDA group exhibited reduced Cleaved Caspase-8, Cleaved Caspase-9, and Bax levels and increased Bcl-2 expression. Together, these results provided important insights, suggesting that co-modification with MSC membrane and PDA prevents Fe_3_O_4_-induced apoptosis in mice brain tissues.

### Effects of NPs on autophagy in brain tissues of mice

**Figure 5:**
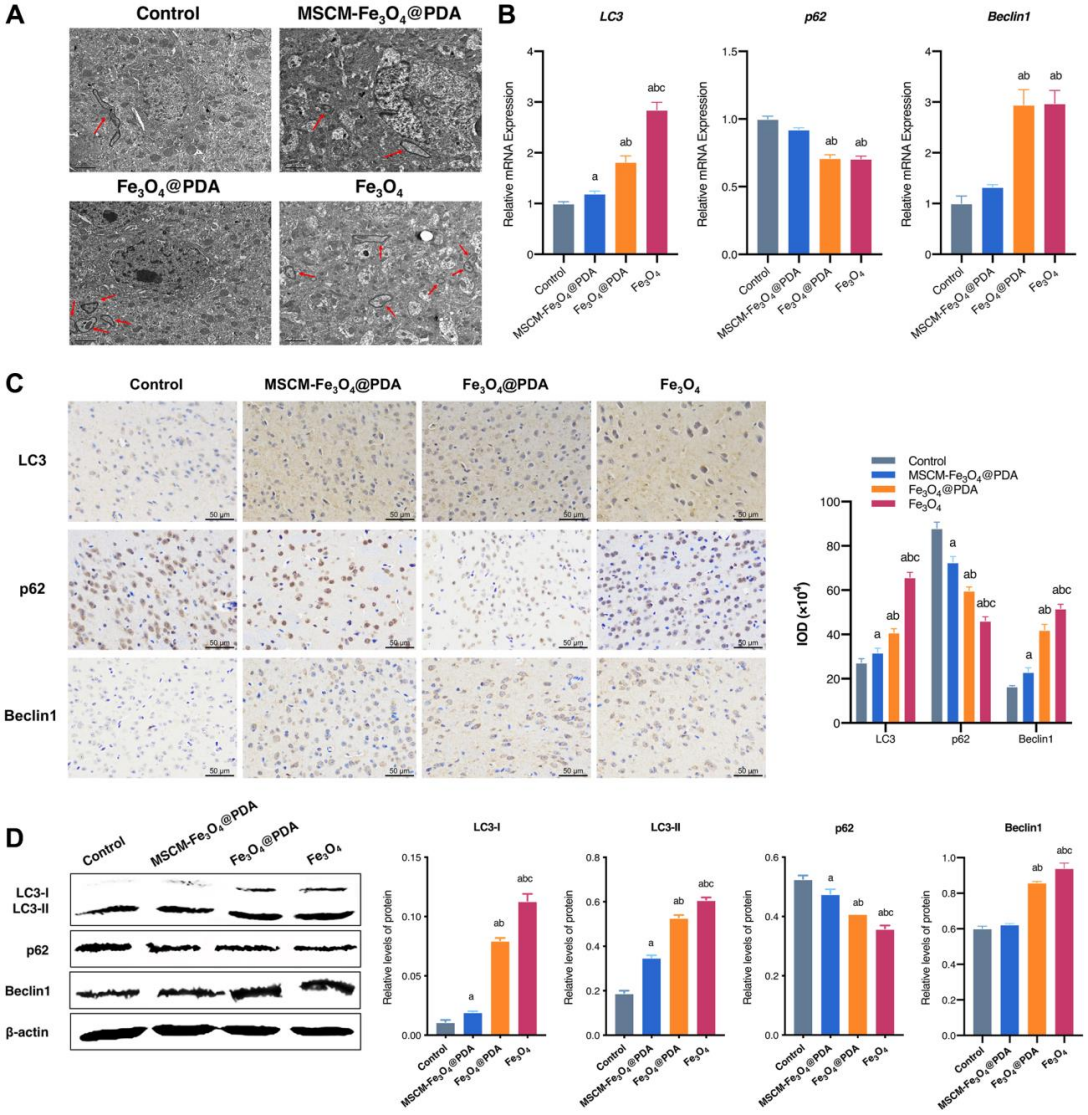
**Effects of MSCM-Fe_3_O_4_@PDA, Fe_3_O_4_@PDA and Fe_3_O_4_ NPs on autophagy.** (**A**) Ultrastructure of autophagy in neurons under electron microscope (autophagy shown by arrow). (**B**) Detection of p62, LC3, and Beclin 1 mRNA levels by RT-qPCR. (**C**) The representative pictures and statistical chart of LC3, p62 and Beclin 1 protein by immunohistochemistry. (**D**) Detection of LC3-I, LC3-II, p62 and Beclin 1 protein by western blot. ^a^*p* < 0.05 when compared with control, ^b^*p* < 0.05 when compared with MSCM-Fe_3_O_4_@PDA, ^c^*p* < 0.05 when compared with Fe_3_O_4_@PDA.

In the present research, autophagy of brain cells was observed by TEM. As shown in Figure 5A, typical autophagic vacuoles accompanied by degradation of cytoplasmic contents were observed in Fe_3_O_4_@PDA and Fe_3_O_4_ treated mice compared with the control and MSCM-Fe_3_O_4_@PDA groups. Next, the effects of MSCM-Fe_3_O_4_@PDA, Fe_3_O_4_@PDA and Fe_3_O_4_ on autophagy in brain tissues of mice were investigated by evaluating the levels of LC3, p62 and Beclin1.

As shown in Figure 5B, the mRNA expression of LC3 and Beclin 1 was significantly increased in the brains of mice exposed to Fe_3_O_4_ NPs. LC3 and Beclin 1 mRNA levels were more significantly decreased in the MSCM-Fe_3_O_4_@PDA group compared with those in the Fe_3_O_4_@PDA group. Furthermore, p62 mRNA expression was significantly decreased in brain tissues of mice exposed to Fe_3_O_4_@PDA and Fe_3_O_4_, and baseline p62 levels were restored by MSC membrane modification. To further verify these results, we assessed p62, Beclin1 and LC3 protein expression by immunohistochemistry (Figure 5C) and western blot (Figure 5D), and the results were consistent with the mRNA levels, suggesting that combined surface functionalization of MSC membranes and PDA reduced the autophagy induced by Fe_3_O_4_ NPs. This finding may be due to the stimulation of mouse brain and the activation of Beclin 1, which adjusts the initiation of autophagy and complex formation with related proteins, thus activating downstream factors to induce autophagy. Extensive crosstalk also occurs between apoptosis and autophagy.

### Effects of NPs on the ASK1/JNK signaling pathway

**Figure 6:**
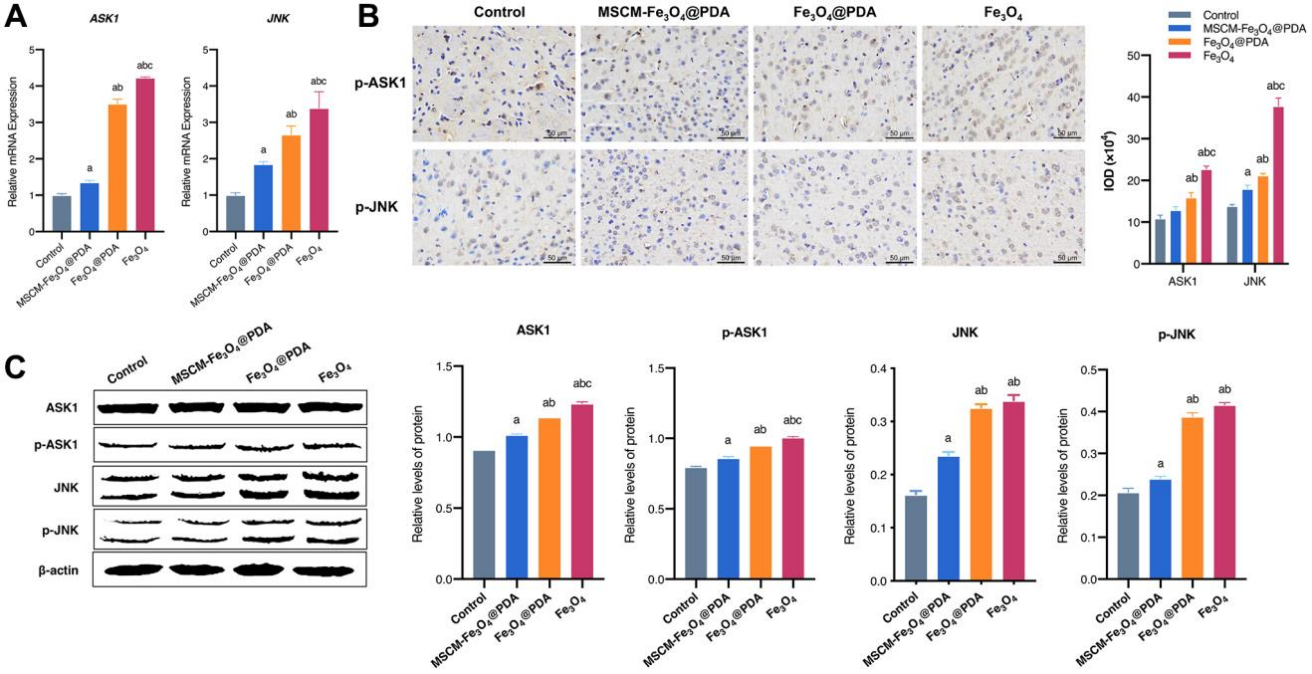
**Effects of MSCM-Fe_3_O_4_@PDA, Fe_3_O_4_@PDA and Fe_3_O_4_ NPs on ASK1/JNK signaling pathway.** (**A**) Detection of ASK1 and JNK mRNA levels by RT-qPCR. (**B**) The representative pictures and statistical chart of p-ASK1 and p-JNK protein by immunohistochemistry. (**C**) Detection of p-ASK1, ASK1, p-JNK and JNK protein by western blot. ^a^*p* < 0.05 when compared with control, ^b^*p* < 0.05 when compared with MSCM-Fe_3_O_4_@PDA, ^c^*p* < 0.05 when compared with Fe_3_O_4_@PDA.

In this experiment, we investigated whether surface functionalization of MSC membrane and PDA could weaken Fe_3_O_4_ NP-mediated inhibition of apoptosis and autophagy by interfering with the ASK1/JNK signaling pathway. RT-qPCR (Figure 6A), immunohistochemistry (Figure 6B) and western blot (Figure 6C) results showed that Fe_3_O_4_ exposure significantly increased ASK1 and JNK mRNA levels and increased total and phosphorylated ASK1 and JNK protein levels in mice brains, while PDA and MSC membrane modification significantly restored ASK1 and JNK levels, especially in the MSCM-Fe_3_O_4_@PDA group.

## DISCUSSION

The excellent superparamagnetism of iron oxide nanoparticles has attracted some attention in molecular science. Nevertheless, the intake of nanoparticles by the organism may cause some toxicity to normal tissues and organs, to name only a few, nanoparticles usually destroy organs through excessive induction of apoptosis and autophagy. The potential mechanism of neurotoxicity of Fe3O4 nanoparticles is summarized in Supplementary Figure 1. The potential therapeutic risk of iron oxide nanoparticles greatly reduces the possibility of their clinical application. In our study, we investigated the MSC membranes’ neuroprotective properties using a mouse model of Fe_3_O_4_ NP-induced neurotoxicity.

Body weight may be influenced by nanoparticles. Zhang et al. found that silver oxide nanoparticles affected rats’ body weight. After receiving an injection of silver oxide nanoparticles, all rats showed significant weight loss and a dose-dependent change [18]. Previously, superparamagnetic Fe_3_O_4_ nanoparticles (SPIONs) have been studied for their effect on body weight in rats. The weight loss was significant for both groups of rats after being fed 50 mg/kg and 100 mg/kg SPIONs for seven days [19]. However, our study found no significant toxic effects on body weight, which might be related to the low exposure dose of NPs. Studies have also shown that inflammatory factors have dual effects on the nervous system, including nutritional protection and neurotoxicity [20]. Therefore, observing changes in inflammatory factors in brain tissues and serum is helpful to understand the neurotoxic mechanism of Fe_3_O_4_ NPs. Microglia and macrophages produce IL-1β and TNF-α, which induce the expression of surface adhesion molecules and other inflammatory mediators [21]. Activated T cells produce IFN-γ which has immunoregulatory effects. IFN-γ synergizes with IL-1β and TNF-α to activate microglia cells [22]. In the chemokine family, MIP-2 belongs to the subfamily C-X-C, and neutrophils are its specific target cells [23]. IL-1β is the main subtype of IL-1 in the brain, and it can stimulate astrocytes and microglia to produce IL-6 and TNF-α [24]. In this experiment, it was found that the levels of IL-1, IL-6 and TNF-α exhibited significant increases, which may indicate a synergistic effect.

MSCs are a type of cells involved in immune regulation and tissue repair. These cells home via chemotaxis to the site of injury and inflammation in the body. These cells control inflammation by regulating neutrophils, macrophages, natural killer cells, dendritic cells and mast cells [25]. Nitric oxide is secreted by MSCs to induce macrophages to produce IL-10 [26]. MSCs also modulate the early stages of inflammation by secreting IL-1 receptor antagonist, in that way blunting the influences of IL-1 and TNF-α on stimulating both aseptic and infectious inflammation [27]. In addition, Zhang et al. found that macrophages acquired an anti-inflammatory M2 phenotype after being cocultured with MSCs, which enhanced wound repair *in vivo* [28]. MSCs have also shown neuroprotective effects, and part of the process can be regulated by IL-6 via the IL-6/STAT3 signaling pathway, enhancing anti-apoptosis signals produced by injured astrocytes [29]. In this study, MSC membrane wrapping materials can reduce brain tissue damage caused by nanoparticles potentially because the stem cell membrane retains its activity and plays an inflammatory repair role.

It is possible that contaminant Fe_3_O_4_ NPs may interact and pass through the blood-brain barrier, causing damage to brain tissues [30]. The toxicity of nanoparticles can be evaluated by detecting ROS and oxidative stress. Neuroinflammation and oxidative stress induced by nerve cell injury are the main toxic effects of nanoparticles [31–33]. When nanoparticles are introduced into the brain, they activate microglia and neurons, producing inflammatory factors such as TNF-α, IL-1β, as well as ROS, which could cause neuronal damage by raising oxidative stress levels in the brain [34]. The ROS, with strong oxidation activity, include superoxide anion, hydroxyl radical, singlet oxygen and hydrogen peroxide. Oxidative stress could be directly reflected by ROS levels. The high levels of ROS can cause damage on biological macromolecules, leading to cell apoptosis or necrosis [35]. MDA is an important peroxide product in the body, with a major impact on the cell metabolism and function, and could reflect the state of oxidative stress [36]. However, antioxidant enzymes, SOD and GSH-Px, for instance, effectively scavenge excess peroxides and free radicals *in vivo*. SOD and GSH-Px activity reflect the function of enzymes to scavenge free radicals and peroxides [37]. Studies have confirmed that the level of oxidative stress would increase and the activity of key antioxidant enzymes (including SOD and GSH-Px) would decrease after long-term injections of Au NPs. Therefore, in comparison with these studies, our research shows that combined surface functionalization MSC membrane and PDA could reduce oxidative stress induced by Fe_3_O_4_ NPs.

Dysregulated cell apoptosis is significant for maintaining tissue homeostasis. In vertebrates, the Bcl-2 protein family plays a significant role in the control and regulation of apoptosis [38]. When apoptosis occurs, Bcl-2 homology 3 (BH3)-only proteins inhibit Bcl-2, and then Bcl-2 antagonist killer 1 (BAK) and Bcl-2-associated X (Bax) proteins are then activated, leading to mitochondrial outer membrane permeabilization. Apoptosis factors are released into the cytoplasm, activating the caspase cascade by activating Caspase-9 [39]. Therefore, similar to caspase levels, the Bcl-2 and Bax also reflect the apoptosis degree. When apoptosis increases, the Bcl-2 protein decreases, and the Bax protein increases. Moreover, caspases play a significant role in inducing apoptosis [40]. Dai et al. used Fe_3_O_4_ and methotrexate (MTX) to treat central nervous system lymphoma. Compared with the MTX monotherapy group, Caspase-3 and Bax expression were upregulated, and Bcl-2 levels were downregulated in the combined nanoparticle treatment group. These findings demonstrated that the thermochemotherapy of Fe_3_O_4_@MTX MNPs might promote apoptosis in diffuse large B-cell lymphoma cells [41]. Wang et al. investigated the effect of actin and Fe_3_O_4_ NPs on the combined treatment of non-small-cell lung cancer. The results showed that nanoparticles increased the level of Bcl-2 and decreased the level of Bax, and the activation of Caspase-3 signaling pathway was observed [42]. The above studies all suggest that the neurotoxicity of nanoparticles may be related to the apoptosis pathway mediated by the BCL-2 family and also the caspase pathway. In our research, compared with the Fe_3_O_4_@PDA group, the MSCM-Fe_3_O_4_@PDA group exhibited the decreased levels of Cleaved Caspase-8, Cleaved Caspase-9, and Bax and the increased expression of Bcl-2. Together, these results provided important insights, suggesting that the combined surface functionalization of MSC membrane and PDA could prevent apoptosis in the brain tissues of mice caused by Fe_3_O_4_ NPs.

As a potential therapeutic target, the regulation of autophagy has attracted increasing attention. Nanomaterials have been recently acknowledged as a novel class of autophagy regulators [42]. However, the effects of nanomaterials on cells differ, and potential mechanisms remain unclear.

Researches have shown that the possible cytotoxic effects of nanoparticles involve autophagy. Zhang et al. found that after the up-conversion of nanoparticles and silica particles were exposed, the autophagy termination process was destroyed in liver cells, indicating that nanoparticles could cause severe liver damage by affecting autophagy [43]. Lou et al. studied the influence of Quercetin nanoparticles on human glioma, demonstrating that Quercetin nanoparticles could promote autophagy and apoptosis by activating LC3/ERK/Caspase-3 and inhibiting the Akt/mTOR signaling pathway [44]. *In vitro* experiments, Li et al. found that silver nanoparticles induced endoplasmic reticulum stress, reduce mammalian mTOR levels, and increase Beclin-1 levels to activate autophagy. *In vivo* experiments, oral administration of silver nanoparticles induced endoplasmic reticulum stress, autophagy and apoptosis in rats, and similar results were noted *in vitro* experiments [45]. These studies were basically consistent with the results of ours. Combined with the relevant results, our results demonstrated that Fe_3_O_4_ NPs could disturb the process of autophagy in brain tissues, and co-modification with MSC membrane and PDA could reduce the occurrence of autophagy, suggesting that MSC membrane-camouflaged nanomaterials exhibit improved biocompatibility. In addition, the enhancement of autophagy activity might be associated with the ROS production induced by Fe_3_O_4_ NPs.

Apoptosis and autophagy are two important functions of cells, and the study of their interaction is a current research hot spot. Signaling molecules, such as JNK, frequently crossover into apoptotic and autophagic signaling pathways, suggesting that JNK has an inevitable relationship with apoptosis and autophagy [46, 47]. ROS activates JNK via numerous mechanisms. The ASK1 pathway serves as a bridge in ROS-mediated JNK pathway activation and can activate autophagy and apoptosis in a ROS-dependent manner [48–50]. ASK1 participates in various reactions, such as inflammation and apoptosis. Earlier studies have demonstrated that ASK1 is activated and responds to ROS, endoplasmic reticulum stress, lipopolysaccharide and other stress responses through the ASK1/JNK pathway and promotes cell apoptosis [51]. ROS contribute to the activation of ASK1 and then JNK in response to oxidative stress, consequently inducing apoptosis [52]. Studies have demonstrated that ASK1 or JNK could trigger off apoptosis after being activated [53, 54]. ASK1/JNK also activates autophagy by modulating Bcl-2 phosphorylation. JNK activated by ROS directly phosphorylates Bcl-2 protein, making Bcl-2 dissociate from Beclin 1, and the Beclin 1-Vps34-PI3K multiprotein complex forms after Beclin 1 is activated, thus activating autophagy [55]. Recent research has shown that exposure to Alumina nanoparticles (Al-NPs) leads to hippocampal-dependent cognitive dysfunction, and combined exposure to Al-NPs and chronic restraint stress aggravates ferroptosis in hippocampal neurons through the activation of IFN-γ/ASK1/JNK signaling pathway [56].

Inhibition of the ASK1/JNK pathway reduces apoptosis and provides a new and comprehensive understanding of the complex mechanisms about tissue protection [57]. What’s more, numerous types of protective substances can regulate apoptosis and autophagy of various cell types through inhibiting the ASK1/JNK signaling pathway, leading to tissue damage [58–60]. Our results showed that MSC membranes significantly reduced ASK1/JNK signaling pathway activation induced by Fe_3_O_4_ NPs, manifesting that the protective effect of MSC membranes on the nervous system of mice might be achieved by alleviating oxidative stress.

## CONCLUSIONS

In summary, our study demonstrated that iron oxide nanoparticles could cause nervous system damage by regulating the ASK1/JNK signaling pathway. Inflammatory aggregation, oxidative stress, apoptosis and autophagy may also play potential roles in this process. Meanwhile, exposure to combined surface functionalization of MSC membrane and PDA showed the neuroprotective effect and may alleviate disorder in the brain nervous system.

## Supplementary Materials

Supplementary Figure 1
