# Supplementary material for: Combined surface functionalization of MSC membrane and PDA inhibits neurotoxicity induced by Fe3O4 in mice based on apoptosis and autophagy through the ASK1/JNK signaling pathway
Source: Aging (Albany NY). 2023 Jul 19;15(14):6933–49. doi: 10.18632/aging.204884 (PMC10415563; doi:10.18632/aging.204884)
Supplement: Supplementary Figure 1 [file aging-15-204884-s001.pdf]

SUPPLEMENTARY FIGURE

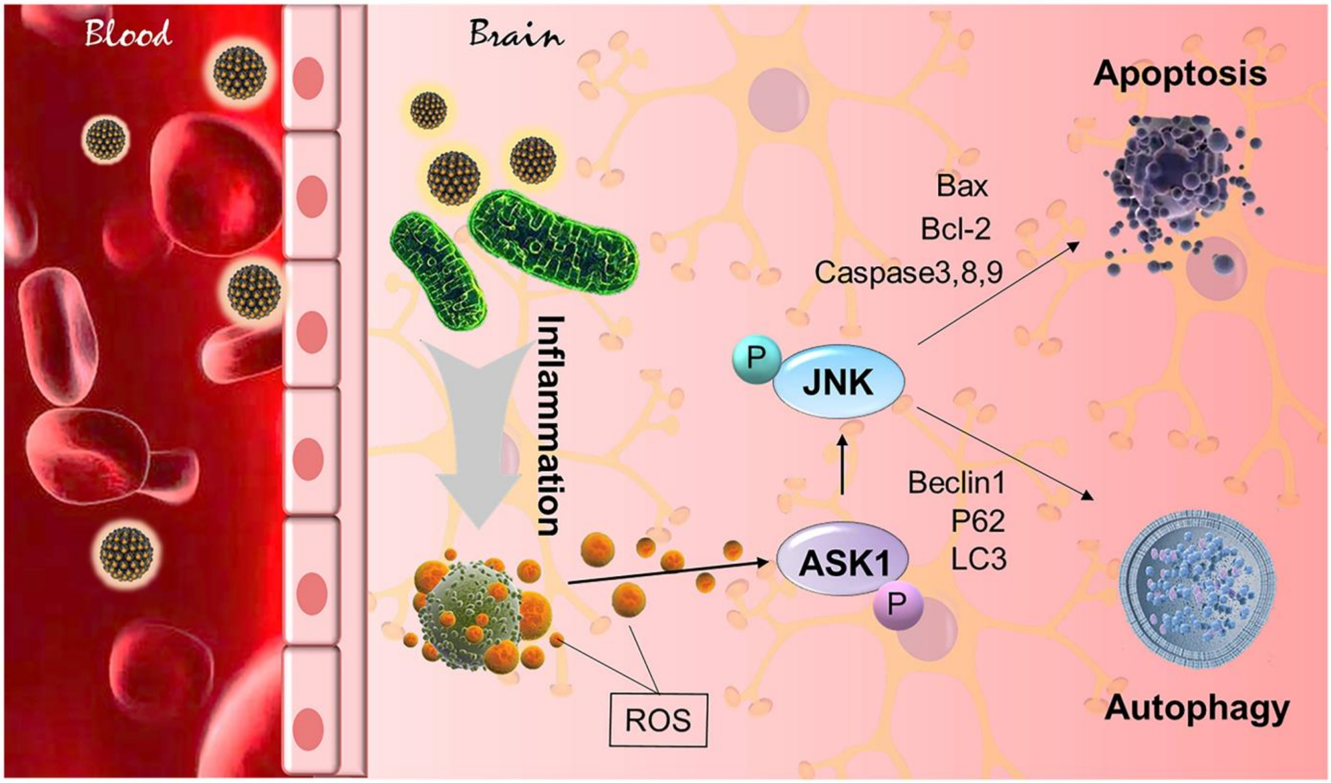

Supplementary Figure 1. The neurotoxicity of Fe_3O_4 nanoparticles. Iron oxide nanoparticles could cause nervous system damage by regulating the ASK1/JNK signaling pathway. Inflammatory aggregation, oxidative stress, apoptosis and autophagy also play potential roles in this process.
